# From theoretical discourse to classroom practice: postcolonial criticism, pedagogical translation, and student reception in multicultural contexts

**DOI:** 10.3389/fpsyg.2026.1821140

**Published:** 2026-07-23

**Authors:** Zhanchen Wan, Xiaoyu Wang

**Affiliations:** 1 Faculty of Education, University of Malaya, Kuala Lumpur, Malaysia; 2 Korean Linguistic and Literature, Sungkyunkwan University (Humanities and Social Sciences Campus), Seoul, Republic of Korea

**Keywords:** intercultural education, literary pedagogy, multicultural classrooms, pedagogical translation, postcolonial criticism, student reception

## Abstract

**Introduction:**

This study explores how postcolonial criticism is pedagogically translated into classroom practice and received by students in multicultural literature classrooms. It examines students’ engagement with five focal postcolonial concepts—otherness, hybridity, mimicry, subalternity, and ambivalence—and considers how their cultural backgrounds and prior understanding of postcolonial criticism shape their reception of these concepts.

**Methods:**

Adopting a qualitative research design, the study collected data over one academic semester through semi-structured interviews and classroom observations. Participants included 10 undergraduate students in literature-related humanities programs, with two each from Malaysia, Japan, South Korea, Russia, and China, together with three university instructors teaching literature and literary theory. Classroom observations were conducted across 12 teaching sessions. Interview transcripts and observational field notes were analyzed thematically, with cross-cultural comparison and triangulation used to identify conceptual difficulties, pedagogical practices, and patterns of student reception.

**Results:**

Students struggled with the complexity of theoretical language and displayed uneven prior understanding of postcolonial criticism’s anti-colonial and anti-Eurocentric foundations. Their understanding also varied across the five concepts: otherness and hybridity were relatively more accessible because students could connect them with experiences of marginality, cultural mixing, and identity negotiation, whereas mimicry, subalternity, and ambivalence required greater conceptual and historical clarification. Localized examples, dialogic teaching methods, and peer perspectives improved the accessibility of theoretical discourse. Reception also varied across cultural contexts: Malaysian and Chinese students connected the theory to identity and marginality; Japanese and South Korean participants showed ambivalence toward its relevance and applicability; and Russian students often resisted its application to their own literary and cultural contexts.

**Discussion:**

The findings highlight the classroom as an interpretive community in which postcolonial theory is not only simplified for pedagogical purposes but also negotiated, contested, and reimagined through cross-cultural interaction. Students’ resistance and ambivalence may therefore function as forms of critical engagement rather than simply as failures of comprehension. These results underscore the need for adaptive pedagogical strategies that balance theoretical rigor, conceptual clarity, historical grounding, and cultural sensitivity in literary education.

## Introduction

Postcolonial criticism has emerged as a central paradigm in literary studies by offering critical vocabularies that interrogate the ways in which literature represents power, identity, and cultural negotiation. In contrast to earlier formalist approaches that privileged esthetic autonomy, postcolonial criticism insists that literary texts are deeply entangled with historical and political processes. By focusing on the intersections between empire, language, and representation, it exposes how narratives of domination and resistance are encoded in literary forms and how they continue to shape interpretive practices across global contexts. Rooted in anti-colonial intellectual traditions, including Fanon’s critique of colonial domination and Said’s critique of Orientalism, postcolonial criticism fundamentally challenges Western, white, Eurocentric universalism and the knowledge hierarchies produced through colonial power. This shift marks not merely a methodological innovation but a transformation in the very purpose of reading and teaching literature, situating interpretation within broader ideological struggles.

The intellectual trajectory of this field underscores its historical depth and continuing vitality. As Young (2016) notes, postcolonial theory not only grapples with the material and psychological legacies of colonialism but also addresses the epistemic hierarchies that structure global systems of knowledge and cultural production. Its insights reach far beyond formerly colonized nations, inviting scholars to reconsider how notions of modernity, civilization, and culture have been discursively constructed. By situating texts within transnational circuits of power and exchange, postcolonial criticism expands the scope of interpretation beyond national canons, creating an analytical framework that links literature to the politics of globalization and identity formation (Bakhronova and Turamuratova, 2024). In this sense, postcolonial criticism is not simply a theory about colonial history; it is also a critical method for examining how power defines whose voices are heard, whose identities are marginalized, and whose knowledge is treated as universal.

Within this broad intellectual landscape, postcolonial theory also presents distinctive pedagogical implications. The same theoretical sophistication that enriches scholarly debates can generate challenges in educational contexts, where students encounter complex terminologies, such as otherness, hybridity, mimicry, subalternity, and ambivalence, that often lack immediate referents in their own cultural experiences. In this study, these five concepts are treated as the focal postcolonial concepts because they capture key dimensions of representation, identity negotiation, imitation, marginal voice, and unstable attachment to dominant cultural authority. When theory is transplanted from the realm of academic discourse into the classroom, the risk arises that it may become an exercise in rote memorization rather than critical engagement. Hence, educators face the task of translating highly abstract theoretical discourses into learning experiences that are both intellectually rigorous and accessible to diverse student audiences.

The tension between conceptual sophistication and pedagogical accessibility underscores a persistent dilemma in literary education. On the one hand, fidelity to theoretical precision demands that students engage with the full complexity of postcolonial ideas; on the other, meaningful learning requires that these ideas be situated within students’ interpretive frameworks. This challenge becomes especially significant when students enter the classroom with uneven prior knowledge of postcolonial criticism’s anti-colonial and anti-Eurocentric foundations. Without this background, students may misrecognize postcolonial theory as another Western theoretical import rather than as a critique of Western epistemic dominance. Bridging this gap calls for pedagogical strategies capable of mediating between theory and experience, between textual abstraction and interpretive praxis. The question is not simply how to simplify theory, but how to cultivate a dialogic classroom environment in which students can actively negotiate meaning and position themselves as participants in the ongoing redefinition of cultural identity.

This pedagogical problem has led to increasing attention to what Andreotti (2011) describes as the “translation” of theory into practice. Pedagogical translation involves processes through which complex theoretical discourses are reframed in ways that invite participation, questioning, and application. Rather than diluting theoretical content, effective translation seeks to open dialogic spaces that enable learners to confront issues of inequality, representation, and cultural hybridity through their own experiences and cultural lenses. For postcolonial pedagogy, such translation also requires teachers to clarify the conceptual meanings of otherness, hybridity, mimicry, subalternity, and ambivalence before asking students to apply them to literary texts. In this sense, the classroom becomes not a site for passive knowledge transmission but a laboratory of intellectual transformation, where theory is enacted and reimagined through situated interactions.

The significance of these pedagogical processes becomes even more pronounced in multicultural classrooms. Here, students from diverse linguistic and cultural backgrounds bring heterogeneous expectations, interpretive repertoires, and epistemological orientations to the study of literature. Such diversity can serve as both an asset and a challenge: it fosters rich dialogic exchange but can also destabilize shared understandings of theoretical terminology. The multiplicity of cultural frameworks complicates the task of achieving conceptual clarity while simultaneously enriching the interpretive possibilities available to the group. Students’ different degrees of familiarity with colonial histories, anti-colonial struggles, and Eurocentric knowledge structures may shape whether they experience postcolonial concepts as relevant, distant, empowering, or imposed. This duality situates multicultural classrooms as vital testing grounds for postcolonial pedagogy.

Moreover, the globalization of higher education has intensified the need to reconsider how theoretical frameworks travel across linguistic and cultural boundaries. When postcolonial theory, originating largely in Anglophone academic traditions, is introduced into classrooms in Asia, Europe, or Africa, it encounters varying institutional histories, literary traditions, and modes of interpretation. The reception of such theory is mediated not only by linguistic translation but also by cultural negotiation and ideological positioning. These encounters reveal that theory is never transferred intact; it is constantly reconstituted through local acts of reading, discussion, and resistance. For example, when students question whether postcolonial criticism applies to Russian, Korean, Japanese, Chinese, or Malaysian literary contexts, such resistance may indicate not only cultural distance but also an incomplete understanding of the theory’s broader critique of colonial and Eurocentric universalism. Understanding these dynamics is crucial for the broader project of decolonizing knowledge production within higher education.

Against this backdrop, the present study investigates how postcolonial criticism is pedagogically translated into classroom practice and how students’ reception reflects processes of negotiation, appropriation, and resistance (Bhabha, 2012). Specifically, the study focuses on five focal postcolonial concepts—otherness, hybridity, mimicry, subalternity, and ambivalence—and examines how students understand, apply, question, or resist these concepts in multicultural literature classrooms. By tracing the transformation of theoretical discourse into interpretive praxis, this research aims to illuminate the mechanisms through which abstract concepts are localized, reinterpreted, and contested in educational settings. The study further examines how students’ prior understanding of postcolonial criticism shapes their reception of these concepts, particularly when they perceive the theory as culturally distant or overly Western. The study thereby contributes to both the field of literary pedagogy and the decolonial agenda in higher education, seeking to articulate how teaching postcolonial theory can become a decolonizing act—one that not only transmits critical knowledge but also reconfigures the epistemic relations between theory, teacher, and learner.

## Literature review

### Postcolonial criticism and contemporary debates

Postcolonial criticism continues to evolve as it negotiates its relationship with emerging intellectual paradigms that challenge the authority of critique itself. Habed (2021) argues that the dialog between postcritique and postcolonial theory has generated a productive tension, compelling scholars to rethink the function of critique in an age marked by affective reading, reparative interpretation, and ethical engagement. In this intersection, postcolonial criticism must balance its long-standing oppositional stance with the affective turn’s emphasis on empathy and connection. The resulting synthesis opens new interpretive possibilities that extend beyond the traditional model of resistance, allowing theory to speak to modes of repair and coexistence.

At the same time, the field’s evolution reveals an enduring concern with maintaining its political edge. Postcolonial theory has historically been grounded in dismantling colonial epistemologies, yet as Habed (2021) notes, it now faces the challenge of preserving this critical rigor while embracing more plural forms of reading. The intellectual project has therefore become twofold: sustaining its deconstructive impulse while developing constructive, dialogic frameworks that reimagine how readers relate to texts and to one another. This duality underscores the field’s vitality and its ongoing struggle to remain relevant in changing academic and cultural landscapes.

Comparative approaches have further expanded postcolonial criticism’s reach. Redondo-Olmedilla (2023) emphasizes that Anglophone and Latin American postcolonial criticism not only differ in genealogy but also in epistemic orientation, illustrating the field’s multiple centers of gravity. The Anglophone tradition often foregrounds discourse, hybridity, and subalternity, while Latin American critics invoke dependency theory, transculturation, and epistemic delinking as key concepts. This diversity highlights that postcolonial criticism is not a monolithic construct but a constellation of locally grounded yet globally connected discourses.

Moreover, the comparative turn reveals the adaptability of postcolonial thought to diverse cultural traditions. Redondo-Olmedilla (2023) suggests that this multiplicity allows the theory to retain relevance across different sociohistorical contexts, reinforcing its potential as a dialogic rather than prescriptive framework. Such pluralism underscores the field’s epistemological resilience—it can inhabit different languages, institutions, and readerships while continuing to interrogate the global dynamics of power and representation. In this sense, postcolonial criticism operates as both a method and a movement: an evolving discourse that continually reconstructs its own boundaries through intercultural encounter and theoretical reinvention.

### Pedagogical translation of literary theory

The question of how abstract literary theory can be translated into pedagogical practice has gained growing scholarly urgency, especially in multilingual and multicultural educational settings. Albrecht (2024) introduces the concept of *pedagogical translation*, describing it as the process through which theoretical content is rearticulated to enhance comprehension in mixed-heritage and L2 classrooms. Rather than simplifying complex concepts, pedagogical translation aligns abstract ideas with learners lived experiences and linguistic repertoires. This approach emphasizes relational understanding—students grasp theory through meaningful association rather than rote memorization.

Pedagogical translation also reframes the teacher’s role from transmitter to mediator of meaning. Albrecht (2024) highlights that educators act as interpreters who construct bridges between academic discourse and students’ interpretive worlds. The effectiveness of this translation depends on dialogic engagement, allowing learners to negotiate theoretical concepts considering their cultural and linguistic identities. Consequently, translation becomes a dynamic process of co-construction, where knowledge emerges through shared inquiry rather than hierarchical instruction.

Building upon this foundation, Pérez Bernardo (2022) situates the pedagogical translation of theory within Spanish-language translation programs. His findings illustrate that theoretical frameworks become intelligible when adapted through localized examples, disciplinary conventions, and linguistic particularities. Students learn to operate abstract ideas by embedding them within culturally resonant cases, thereby internalizing theory through application. This pedagogical process transforms theoretical discourse into an experiential practice that empowers students to engage critically and contextually.

Knights (2017) extends the discussion by proposing dialogic teaching models that position the classroom as a site of negotiation rather than transmission. Through dialogic pedagogy, theory is not merely taught but enacted students and instructors collaboratively test, revise, and reinterpret conceptual frameworks through discourse.

The classroom thus functions as a microcosm of critical inquiry, where theoretical meaning is co-authored through dialog. This perspective aligns pedagogical translation with broader constructivist approaches, affirming that theory attains pedagogical efficacy only when it becomes a living, dialogic practice.

### Decolonizing pedagogy and methodologies

The effort to align postcolonial theory with educational practice often converges with broader movements toward decolonizing knowledge. Andreotti (2011) underscores that postcolonial ideas must move beyond theoretical abstraction to become actionable pedagogical resources. She conceptualizes *actionable postcolonial theory* as an approach that empowers learners to interrogate global inequalities and reconstruct their epistemic positions within the classroom. This model redefines education as a transformative act—one that connects theory to ethical responsibility and social change.

Andreotti’s framework positions the classroom as a decolonial space where learners examine their own positionalities within structures of privilege and marginalization. Decolonizing pedagogy, in this view, is not a mere adaptation of Western theories but an epistemic intervention that seeks to democratize knowledge production. The goal is to cultivate what she terms “critical literacy,” enabling students to recognize and contest the ideologies embedded in texts, institutions, and even pedagogical methods. By transforming awareness into agency, decolonizing pedagogy transforms learning into a political and ethical endeavor.

Tuhiwai Smith (2021) extends this argument through her influential articulation of *decolonizing methodologies*. She emphasizes that both research and teaching must dismantle the epistemic hierarchies that privilege Western modes of knowing while marginalizing indigenous and local epistemologies. Her framework reorients inquiry toward relationality, reciprocity, and respect for community knowledge. When applied to pedagogy, these principles challenge educators to design curricula that value multiple epistemologies and center marginalized perspectives, ensuring that the classroom itself becomes a site of epistemic justice.

Mukherjee (2019) complements these perspectives by invoking *Southern Theory* within comparative education, demonstrating how postcolonial frameworks can unsettle Eurocentric models of knowledge. By drawing from intellectual traditions of the Global South, Mukherjee argues for a decolonial comparative paradigm that foregrounds plurality and situatedness. Together, these contributions articulate a coherent vision: decolonizing pedagogy is both a theoretical and methodological project that transforms the politics of knowledge, repositioning the classroom as a dynamic site of critical resistance and epistemic renewal.

### Student reception and intercultural competence

Understanding how students receive, interpret, and internalize literary theory is critical for evaluating pedagogical effectiveness. Rosenblatt’s (1994) transactional theory offers a foundational lens, conceptualizing reading as a reciprocal process between text and reader. Meaning, in this view, arises through transaction rather than extraction: the act of reading becomes an event shaped by the reader’s emotions, prior knowledge, and sociocultural background. This framework has profoundly influenced the study of student reception, foregrounding the interpretive agency of learners as co-creators of textual meaning.

Building on Rosenblatt’s foundational insights, contemporary studies highlight that reception is not only interpretive but also intercultural. Mardones et al. (2024) develop a didactic design that integrates multimodal reading strategies with the cultivation of intercultural competence. Their findings suggest that comprehension in diverse classrooms extends beyond textual decoding to include empathy, perspective-taking, and cross-cultural awareness. In this model, theoretical learning is inseparable from intercultural negotiation: understanding a concept like “hybridity” requires navigating multiple cultural logics simultaneously.

Labanieh (2022) further complicates the picture by analyzing how humor, irony, and translatability shape students’ reception of world literature. She demonstrates that comprehension gaps often stem from cultural untranslatability rather than intellectual difficulty, indicating that reception is mediated by linguistic and cultural positioning. Recognizing this complexity, effective pedagogy must acknowledge the uneven terrain of accessibility across cultural contexts, fostering reflective spaces where misunderstanding becomes a productive site for intercultural learning.

Finally, integrating these insights suggests that student reception is best understood as a dynamic process negotiated at the intersection of text, theory, and cultural context. Rather than treating comprehension as a fixed outcome, scholars now view it as an ongoing dialog shaped by affect, identity, and power relations. The implication for literary pedagogy is profound: classrooms must be designed not only to transmit theoretical knowledge but to cultivate the intercultural competencies that make such knowledge meaningful. In this sense, reception becomes an ethical and transformative dimension of learning, aligning with the broader goals of postcolonial and decolonizing education.

## Methodology

### Research design

This study adopts a qualitative research design aimed at capturing the complexity of how postcolonial criticism is pedagogically translated into classroom practice and how students from different cultural backgrounds receive and interpret theoretical discourse. Qualitative inquiry is particularly appropriate for this project because it privileges depth over breadth, allowing for an exploration of the interpretive, dialogic, and context-dependent nature of student and teacher experiences. By sitting in the classroom as a site of meaning negotiation, the study positions itself within interpretive traditions that foreground the lived realities of learners in multicultural contexts.

### Participants

The study recruited a total of 10 undergraduate students majoring in the humanities, specifically literature-related programs, across diverse national backgrounds. The sample was distributed evenly, with two participants each from Malaysia, Japan, South Korea, Russia, and China. This configuration was chosen to reflect a range of linguistic, cultural, and educational repertoires, ensuring that the study would capture cross-cultural patterns of reception while avoiding the dominance of any single national perspective. In addition, three university instructors who teach literature and literary theory were included in the sample: two based in Malaysia and one based in South Korea. The inclusion of both students and teachers provides a two-dimensional perspective, highlighting not only how theory is received but also how it is framed and mediated through pedagogical practices.

### Data collection

Data collection was conducted over the course of a single academic semester and followed a two-phase, two-country design to ensure cultural and contextual diversity while maintaining methodological consistency. The same semi-structured interview guide and classroom observation protocol were used in both phases.

In Phase 1, conducted in Malaysia, data were gathered from Malaysian and Chinese undergraduate students enrolled in literature and humanities programs at two public universities. In Phase 2, carried out in South Korea, participants included Japanese, Korean, and Russian students from comparable academic programs at a leading comprehensive university. This cross-national sampling strategy allowed the study to capture variations in interpretive engagement across linguistic and cultural backgrounds while controlling for disciplinary context.

Semi-structured interviews constituted the primary source of qualitative data. Each of the 10 student participants engaged in an in-depth, one-on-one interview lasting approximately 60–75 min. The interviews explored students’ initial encounters with theoretical vocabulary, their perceived difficulties in grasping key concepts such as *otherness*, *hybridity*, and *mimicry*, and the strategies they used to construct meaning. Open-ended prompts invited participants to reflect on their interpretive experiences rather than provide evaluative judgments. Follow-up questions were used to probe specific classroom episodes, prompting students to recall moments when they felt either confusion or insight. Interviews were conducted primarily in English, with occasional code-switching to participants’ first languages when clarification was needed. All interviews were audio-recorded with prior consent and transcribed verbatim for detailed thematic coding.

Teacher interviews complemented the student data by revealing the instructional perspective underlying classroom practices. Three instructors, two from Malaysian universities and one from a South Korean university, participated in extended interviews lasting 75–90 min. The discussions focused on their pedagogical philosophies, approaches to simplifying complex theoretical discourse, and reflections on intercultural communication within multilingual classrooms. Teachers were asked to provide concrete examples of classroom moments that illustrated successes or challenges in teaching postcolonial concepts. These interviews yielded rich accounts of how educators balanced theoretical fidelity with accessibility, and how they understood the process of *pedagogical translation* as an act of negotiation between abstract knowledge and lived cultural context.

To triangulate the interview findings, a systematic program of non-participant classroom observation was conducted across 12 teaching sessions, evenly distributed throughout the semester. Each session lasted approximately 90 min, and observation centered on the introduction and discussion of key theoretical terms such as *subaltern*, *hybridity*, and *ambivalence*. The researcher adopted an unobtrusive stance, taking detailed field notes that recorded the sequence of teacher explanations, the nature of student responses, and the rhythm of classroom dialog. Attention was also paid to nonverbal indicators, pauses, shifts in tone, and body language, that signaled moments of comprehension, hesitation, or resistance. These observations provided a real-time view of how theory was enacted, contested, and collectively interpreted *in situ*.

The integration of interviews and observations formed an iterative cycle of analysis. Whenever students referred in interviews to specific classroom episodes, the corresponding observation notes were revisited to reconstruct the interactional context. This cross-referencing allowed the identification of recurring pedagogical patterns such as simplification through local examples, reinterpretation through peer dialog, and collaborative attempts to stabilize theoretical meaning. In this way, each data source illuminated the other, producing a layered and reflexive account of how theoretical discourse circulates within multicultural learning environments.

All stages of data collection were conducted under a rigorous ethical framework. Prior to commencement, ethical approval was obtained from the University of Malaya Research Ethics Committee (UMREC). Participants were fully briefed about the voluntary nature of participation, confidentiality measures, and their right to withdraw at any time without penalty. Written informed consent was obtained from all participants before interviews or observations. All identifying details were removed during transcription, and pseudonyms were used in subsequent analysis. Audio recordings and field notes were stored on encrypted institutional drives accessible only to the research team. This adherence to ethical and data-protection standards not only safeguarded participants’ rights but also enhanced the study’s methodological transparency and credibility.

### Research procedure

**Figure 1:**
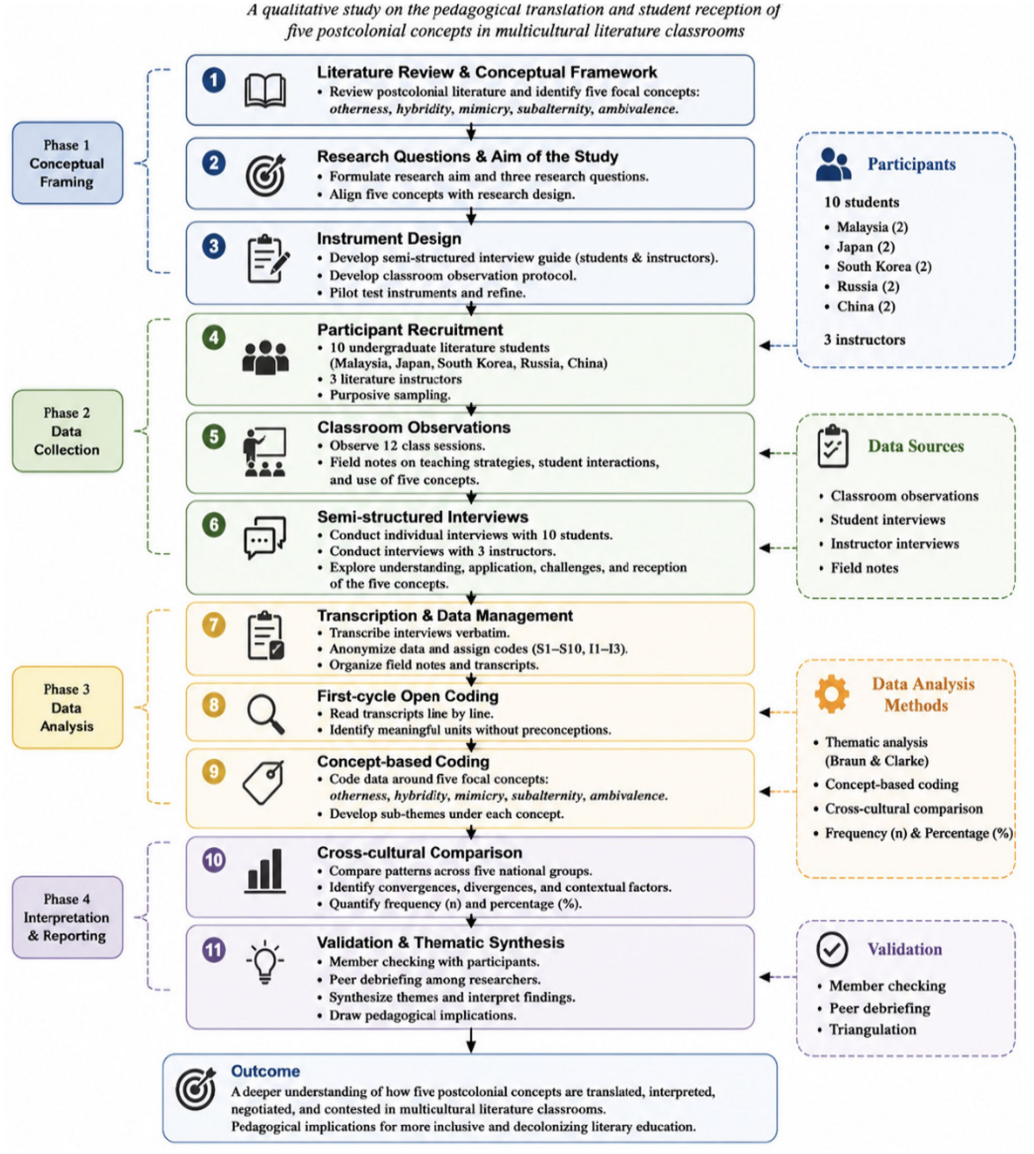
Research procedure.

To enhance the transparency and replicability of the qualitative research design, the study followed a systematic procedure that connected conceptual framing, data collection, data analysis, and interpretive validation. As shown in Figure 1, the research process began with a review of postcolonial literature and the identification of five focal concepts: otherness, hybridity, mimicry, subalternity, and ambivalence. These concepts informed the formulation of the research aim, research questions, interview guide, and classroom observation protocol. The study then recruited 10 undergraduate literature students from Malaysia, Japan, South Korea, Russia, and China, together with three literature instructors. Data were collected through classroom observations, semi-structured interviews, and field notes, allowing the study to capture both instructional practices and students’ reception of postcolonial criticism in multicultural classroom contexts.

Figure 1 illustrates that the analysis proceeded through several connected stages. After transcription and anonymization, the data were first examined through open coding to identify recurring meanings without imposing predetermined categories. The second stage used concept-based coding to examine how participants understood and responded to the five focal postcolonial concepts. The study then conducted cross-cultural comparison to identify similarities and differences across national groups, with frequencies and percentages calculated where appropriate to support the interpretation of patterns. Finally, member checking, peer debriefing, and triangulation were used to strengthen trustworthiness. This step-by-step procedure ensured that the findings were not derived from isolated interview impressions but from a transparent and traceable analytic process linking theoretical concepts, classroom practices, student responses, and pedagogical implications.

### Data analysis

All interviews were audio-recorded, transcribed verbatim, and analyzed alongside field notes using thematic analysis. The coding process was inductive but guided by sensitizing concepts derived from postcolonial criticism, such as hybridity, mimicry, and otherness. First-cycle open coding was employed to identify recurring themes in both teacher and student accounts, followed by axial coding to establish connections between pedagogical strategies and student reception. Particular attention was given to intercultural comparisons, tracing how students from different national backgrounds negotiated similar concepts and how teachers’ strategies either facilitated or complicated understanding. Through iterative analysis, data were organized into thematic clusters that illuminated the relationship between theoretical discourse, pedagogical translation, and reception experiences.

### Trustworthiness and ethics

To ensure trustworthiness, several strategies were employed. Data triangulation was achieved by drawing on interviews from both teachers and students as well as observational notes. Member checking was conducted by returning preliminary interpretations to selected participants for feedback and validation. Peer debriefing with colleagues in literary pedagogy and cultural studies further enhanced interpretive rigor. Ethical approval was obtained prior to data collection, and all participants provided informed consent. Anonymity was maintained by replacing real names with pseudonyms and ensuring that identifying details about institutions or courses were withheld.

## Results

Thematic analysis of interview data identified four overarching themes that collectively illustrate how postcolonial theory is mediated, translated, and received in multicultural classroom settings. As shown in Table 1 (as below), each theme encompasses several sub-themes that capture both cognitive and pedagogical dimensions of learning, as well as the intercultural negotiations embedded in theoretical interpretation. The themes emerged through iterative coding of teacher and student interviews, triangulated with classroom observations, and reflect the multidimensional processes through which theoretical abstraction becomes pedagogical practice.

**Table 1:** Operational definitions of the five postcolonial concepts.

Concept	Definition in postcolonial context	Key theoretical orientation
Otherness	The discursive construction of colonized, racialized, or culturally different groups as inferior, marginal, or external to the dominant self	Said; colonial discourse
Hybridity	The formation of mixed cultural identities, meanings, and practices through colonial and intercultural encounters	Bhabha
Mimicry	The ambivalent imitation of dominant colonial or metropolitan norms by colonized subjects, producing both resemblance and disruption	Bhabha
Subalternity	The condition of structurally marginalized subjects whose voices are excluded or mediated by dominant systems of representation	Spivak; Gramsci
Ambivalence	The unstable coexistence of attraction and resistance toward colonial authority, dominant culture, or imported theoretical frameworks	Bhabha

Table 2 shows the four major themes collectively depict the classroom as a site of epistemic negotiation where abstract theoretical language is continually mediated by cultural experience and pedagogical practice. Students’ initial struggles with terminology and conceptual density underscore the challenges of translating postcolonial theory into actionable understanding. However, the emergence of localized teaching strategies and dialogic participation demonstrates that comprehension develops not through simplification alone, but through situated engagement. Teachers’ adaptations reveal that theory acquires meaning when reframed within learners’ cultural frames of reference, validating the notion of “pedagogical translation” as a creative act of re-theorization.

**Table 2:** Thematic framework of student and teacher perspectives on postcolonial theory.

Main themes	Sub-themes	Illustrative focus (from interviews)
Comprehensibility of theoretical discourse	Difficulty with abstract terminology	Students frequently reported that concepts such as *hybridity* and *mimicry* were overly abstract and difficult to connect with specific literary texts
	Partial understanding through personal experience	Some students were able to associate ideas like *otherness* with their own cultural positions, though they struggled to apply them in an academic context
	Teachers’ strategies of simplification	Teachers often relied on examples, analogies, and translations to make complex theoretical concepts more accessible
Pedagogical translation practices	Localizing theory through contextual texts	Teachers observed that when using local or familiar literary works, students found it easier to grasp theoretical concepts
	Dialogic and participatory teaching	Classroom discussions and dialogic teaching strategies helped students negotiate meaning collaboratively
	Constraints of curriculum and time	Teachers acknowledged that limited time and curricular demands restricted the depth of theoretical explanation
Student reception in multicultural contexts	Cross-cultural resonance and resistance	Students from different national backgrounds showed varying degrees of acceptance and resistance toward postcolonial concepts
	Negotiating identity through theory	Students used concepts such as *otherness* to reflect on their own cultural identities, though the depth of reflection varied
	Perception of theory’s universality	Some students questioned whether postcolonial theory was universally applicable, especially to non-Western literary traditions
Classroom as interpretive community	Collaborative meaning-making	Interaction between teachers and students enabled fragmented understandings to be transformed into shared interpretations
	Role of peer perspectives	Cross-cultural peer exchanges encouraged students to recognize and appreciate diverse cultural interpretations of postcolonial theory

Themes and sub-themes were derived through thematic analysis of interview transcripts with students and teachers. Illustrative focus excerpts are paraphrased or shortened from participants’ original statements to protect anonymity and enhance readability.

Equally significant are the findings related to student reception and the role of the classroom as an interpretive community. Students’ negotiations between acceptance and resistance reflect a dynamic process of intercultural meaning-making in which postcolonial theory functions simultaneously as a lens and a mirror. Through peer dialog and collaborative interpretation, learners transformed theoretical abstraction into shared understanding, highlighting the classroom’s role as both a cognitive and social space for decolonizing thought. These results affirm that the pedagogical vitality of postcolonial criticism lies not in its stability, but in its openness to continual reinterpretation within diverse cultural contexts.

### Comprehensibility of theoretical discourse

#### Difficulty with abstract terminology

Students across different national backgrounds consistently described postcolonial theory as linguistically dense and conceptually abstract. Terms such as *hybridity*, *mimicry*, and *ambivalence* were identified as particularly difficult to understand, as their meanings did not seem immediately apparent from the words themselves. Several participants explained that, although they could memorize the definitions provided by teachers, they struggled to internalize the concepts or apply them to the analysis of specific texts. This gap between knowing terminology and using it effectively revealed the ways in which theory can appear distant from the practical needs of learners in the classroom.

The problem was further compounded in a multicultural setting where students’ linguistic repertoires varied. Non-native speakers of English highlighted the additional difficulty of encountering specialized theoretical vocabulary in a second language. One Japanese student noted that *“it feels like another layer of translation beyond the text itself,”* which hindered comprehension. These findings suggest that terminology functions as both a cognitive and linguistic barrier, reinforcing the perception that postcolonial theory belongs more to the domain of experts than to learners.

#### Partial understanding through personal experience

Despite the difficulty with terminology, some students reported finding points of connection between theoretical concepts and their personal or cultural experiences. For example, Malaysian and Chinese participants linked the notion of *otherness* to their own sense of being positioned as outsiders in academic or social contexts. As one student explained, *“I understand otherness because I have sometimes felt it myself in this classroom.”* By drawing on these lived experiences, students were able to give the concept a tangible meaning, even if they could not fully articulate it within the conventions of academic discourse.

However, this kind of experiential linkage was uneven across the sample. While certain students found postcolonial concepts empowering for reflecting on issues of identity and cultural hierarchy, others dismissed them as too far removed from their immediate realities. A Russian student remarked that *“this theory feels too Western, and I do not think it really applies to Russian literature.”* These divergences highlight the uneven applicability of theory across cultural contexts and reveal the limits of relying solely on personal resonance as a pathway to comprehension.

#### Teachers’ strategies for simplification

Teachers demonstrated awareness of students’ struggles and employed a variety of strategies to make postcolonial theory more accessible. One common approach was the use of culturally familiar examples, where instructors connected abstract terms such as *hybridity* to local texts or popular cultural references. As a Malaysian teacher observed, *“when I use a local novel or short story, students immediately engage with the idea of hybridity.”* By anchoring theoretical concepts in familiar narratives, teachers created a bridge between abstract discourse and the interpretive frameworks that students already possessed.

Another strategy involved breaking down theoretical concepts into smaller components and guiding students through them gradually. Teachers described designing classroom activities where students practiced applying one concept at a time to specific passages before attempting more complex analyses. A Korean instructor reflected that *“if we take one step at a time, students feel less intimidated and are more willing to experiment with the terms.”* This scaffolded approach encouraged incremental engagement with theory, reducing the sense of intimidation associated with unfamiliar terminology while promoting sustained interaction with abstract discourse.

### Pedagogical translation practices

#### Localizing theory through contextual texts

Teachers frequently emphasized the importance of using locally relevant or culturally familiar texts to facilitate students’ engagement with postcolonial theory. By anchoring abstract concepts in narratives closer to students lived realities, instructors attempted to bridge the gap between theoretical language and experiential understanding. One Malaysian teacher observed that *“when I introduce hybridity through a Malaysian short story, students suddenly realize the concept is not only about distant colonial histories but also about their everyday encounters with cultural mixing.”* This suggests that localization provides students with a concrete point of entry into otherwise abstract theoretical frameworks.

Students, in turn, often confirmed the effectiveness of this approach. A Chinese participant explained that *“when the teacher used an Asian novel instead of only Western texts, I felt the theory was more relevant to me.”* Such testimonies highlight how localizing theory does not merely simplify difficult ideas but also validates students’ cultural identities as part of the interpretive process. In multicultural classrooms, this strategy appears especially valuable, as it offers diverse groups of students the opportunity to test the applicability of theory across varied cultural and literary contexts.

#### Dialogic and participatory teaching

Another pedagogical strategy reported by both students and teachers involved creating dialogic and participatory classroom environments. Teachers encouraged open-ended discussions where students could experiment with theoretical vocabulary and articulate their interpretations without fear of immediate correction. A South Korean instructor reflected that *“if we let students talk through the concepts, even with mistakes, they begin to make sense of the theory in their own way.”* Such dialogic practices enabled students to co-construct knowledge and lowered the barrier between authoritative theory and lived interpretation.

Students expressed appreciation for these interactive spaces, particularly in contrast to lecture-based approaches. One Japanese student commented, *“when I hear my classmates explain otherness, I can compare it with my own idea and sometimes it makes the meaning clearer.”* The evidence suggests that dialogic pedagogy fosters collaborative meaning-making, allowing students to learn not only from teachers but also from their peers’ diverse cultural perspectives. In this sense, the classroom becomes a collective site of negotiating theoretical discourse rather than a unidirectional transmission of knowledge.

#### Constraints of curriculum and time

Despite the creative strategies teachers employed, structural constraints limited the extent to which postcolonial theory could be fully explored in the classroom. Instructors frequently mentioned that curricular demands and limited instructional time forced them to condense complex theoretical ideas into simplified forms. One Malaysian teacher admitted, *“I know the students need more time with these concepts, but the syllabus pushes us to move quickly.”* Such pressures often reduce opportunities for deeper exploration and iterative application of theory.

Students were aware of these limitations as well. A Russian student noted that *“sometimes the class just touches the theory and then moves on; it feels unfinished.”* These accounts reveal the tension between pedagogical intent and institutional constraints. While teachers recognized the need for sustained engagement, external requirements often curtailed the process, leaving both teachers and students with a sense of partial understanding.

### Student reception in multicultural contexts

#### Cross-cultural resonance and resistance

Students from different cultural backgrounds demonstrated varying levels of resonance with postcolonial concepts. For some, the framework offered a powerful tool to articulate experiences of marginality. A Malaysian student shared that *“when we talked about otherness, I felt it described exactly what I have experienced studying abroad.”* This indicates that theory can function as a lens through which students reflect on their lived realities, fostering a sense of recognition and validation.

In contrast, other students expressed resistance, perceiving postcolonial concepts as disconnected from their own traditions. A Russian participant remarked, *“postcolonialism is not our history, so I do not see why it should apply to Russian writers.”* Such responses highlight how the reception of theory is mediated by students’ cultural and historical contexts, which may either facilitate or constrain their willingness to engage with its ideas.

#### Negotiating identity through theory

For many students, the encounter with postcolonial criticism triggered reflection on their own identities. A Chinese student explained, *“the idea of hybridity makes me think about how I use both Chinese and Western frameworks in my studies.”* This suggests that theoretical concepts can open productive spaces for self-examination, enabling students to interrogate their positions within broader cultural and academic systems.

However, this negotiation of identity was not always straightforward. Some students experienced discomfort when theory challenged their established perspectives. A Japanese student admitted, *“sometimes I feel the theory pushes me to question my culture in ways I am not ready for.”* Such moments of tension reveal the ambivalence of reception: while postcolonial theory can empower critical self-reflection, it can also unsettle students’ sense of cultural stability.

#### Perception of theory’s universality

Another recurring theme concerned students’ views on the universality, or lack thereof, of postcolonial theory. Several participants questioned whether concepts developed in Western academic contexts could be applied globally. A South Korean student asked, *“is hybridity really useful for understanding Korean literature, or is it just a Western idea we have to adopt?”* This reflects skepticism about the adaptability of theoretical frameworks across cultural and literary boundaries.

At the same time, some students argued that the strength of postcolonial theory lies precisely in its adaptability. A Malaysian participant stated, *“if we reinterpret the concepts in our own way, they can work for any literature.”* These divergent perspectives suggest that the classroom becomes a site where the universality of theory is tested, negotiated, and sometimes redefined, depending on how students integrate it with their cultural reference points.

### Classroom as interpretive community

#### Collaborative meaning-making

The classroom often functioned as a collective space where fragmented understandings of theory were transformed into shared interpretations. Teachers encouraged students to collaboratively analyze passages using concepts such as *otherness* or *hybridity*, and through group dialog, individual uncertainties were clarified. A Malaysian teacher noted, *“when students talk in small groups, their ideas start to connect, and suddenly the concept looks clearer to them.”* This highlights how meaning emerges through interaction rather than isolated study.

Students echoed the value of these collaborative processes. One Japanese participant reflected, *“sometimes I do not understand the theory until I hear my classmates explain it in their own words.”* Such accounts reveal that interpretive communities allow students to borrow, adapt, and refine one another’s insights, producing a richer understanding of postcolonial criticism than could be achieved individually.

#### Role of peer perspectives

Peer perspectives played a critical role in shaping how students engaged with theoretical discourse. For many, hearing interpretations from classmates of different cultural backgrounds illuminated aspects of theory they had not considered. A South Korean student explained, *“when a Russian classmate described hybridity with her own cultural examples, I realized the concept can work in many ways.”* This underscores the importance of cross-cultural dialog in broadening the interpretive scope of theoretical learning.

At the same time, peer perspectives also created opportunities for students to challenge each other’s assumptions. A Chinese participant recalled, *“I thought mimicry was only about colonizers and colonized, but my Malaysian friend said it also fits with language learning, which made me think differently.”* These exchanges reveal the dynamic potential of multicultural classrooms, where peer contributions not only complement but also complicate dominant readings of theory.

#### Tensions and misalignments

While collaborative and peer-driven approaches enriched classroom discussions, they also introduced tensions. Differences in cultural backgrounds sometimes led to divergent interpretations that could not be easily reconciled. A Russian student shared, *“I argued with others because I felt postcolonialism does not belong to our history, but they insisted it is universal.”* Such disagreements illustrate how classrooms can become contested spaces where theoretical universality is both affirmed and resisted.

Teachers observed similar moments of dissonance, which they interpreted as both challenges and learning opportunities. A South Korean instructor reflected, “*students do not always agree, but in the debate, they discover the limits of the concepts and also their possibilities*.” These findings suggest that misalignments should not be viewed solely as obstacles but as productive tensions that enable students to critically negotiate the scope and relevance of postcolonial theory.

### Summary of findings

Cross-cultural comparison revealed that while students shared some common struggles with postcolonial theory, their interpretations and receptions were shaped by distinct cultural and historical contexts. To highlight these differences and convergences, the table below synthesizes key perspectives from students across five national backgrounds.

Table 3 suggests that Malaysia and China displayed relatively higher receptivity to postcolonial concepts, as students connected theory with personal experiences of marginality and hybridity. In both cases, localized examples offered by teachers further reinforced the relevance of theoretical discourse, enabling students to adapt the framework to their own contexts.

**Table 3:** Comparative perspectives of students from different national backgrounds.

Country	Key perspectives on postcolonial theory
Malaysia	Saw relevance in terms such as *otherness* when linked to personal experiences of marginality; valued use of local texts in class; viewed theory as adaptable if reinterpreted
Japan	Reported difficulty with abstract terminology; appreciated peer explanations during discussions; sometimes felt unsettled by theory’s challenge to cultural identity
South Korea	Questioned whether concepts like *hybridity* were directly applicable to Korean literature; recognized the usefulness of dialogic and peer-based learning; emphasized adaptability of theory when localized
Russia	Expressed skepticism, arguing that postcolonial theory reflects Western experiences; often resisted its application to Russian literary contexts; highlighted unfinished engagement due to curriculum constraints
China	Related to the concept of *otherness* in terms of academic and cultural positioning; acknowledged hybridity as part of intellectual practice; questioned universality while seeking culturally specific adaptations

In contrast, Japan and South Korea showed a more ambivalent reception. While Japanese students benefited from dialogic discussions and peer exchanges, they also expressed discomfort when theory unsettled their cultural assumptions. South Korean students questioned the applicability of Western-derived concepts but acknowledged that theory could be reinterpreted through local perspectives, demonstrating both resistance and flexibility.

Russia stood out as the most resistant case, with students consistently viewing postcolonial theory as disconnected from their cultural and literary histories. Their skepticism highlighted the limits of theoretical universality and the challenge of applying postcolonial discourse in contexts without direct colonial legacies. However, this resistance also provided critical tension, drawing attention to the necessity of adapting theory to diverse literary traditions.

Together, these findings underscore the dual nature of postcolonial theory in multicultural classrooms: while it can serve as a powerful interpretive lens that fosters identity reflection and critical awareness, it also exposes cultural fault lines that complicate its reception. The classroom thus becomes a space where theory is simultaneously validated, contested, and reimagined through cross-cultural encounters.

Table 4 summarizes how students understood and applied the five focal postcolonial concepts, as well as how strongly instructors emphasized each concept in classroom teaching. The results show that otherness was the most accessible concept for students, with only 4 of 10 students (40%) reporting difficulty and 7 of 10 students (70%) able to apply it to their own cultural or learning contexts. This suggests that students could more easily understand otherness when it was connected to experiences of marginality, exclusion, or outsider positioning. Hybridity was also relatively accessible, although half of the students (50%) still reported difficulty. Six students (60%) were able to apply the concept through examples of cultural mixing, bilingual experience, and identity negotiation.

**Table 4:** Students’ understanding of five postcolonial concepts.

Concept	Main pattern of understanding	Students reporting difficulty *n* (%)	Students showing contextual application *n* (%)	Instructor emphasis *n* (%)
Otherness	Easier to understand when linked to marginality and outsider experience	4/10 (40%)	7/10 (70%)	3/3 (100%)
Hybridity	Understood through cultural mixing, bilingualism, and identity negotiation	5/10 (50%)	6/10 (60%)	3/3 (100%)
Mimicry	Often confused with simple imitation; clearer after textual examples	7/10 (70%)	4/10 (40%)	2/3 (66.7%)
Subalternity	Most difficult due to abstract relation between voice, power, and representation	8/10 (80%)	3/10 (30%)	2/3 (66.7%)
Ambivalence	Understood as mixed feelings toward theory, culture, or authority	6/10 (60%)	5/10 (50%)	2/3 (66.7%)

By contrast, mimicry and subalternity presented greater conceptual challenges. Seven students (70%) had difficulty with mimicry, often reducing it to simple imitation before instructors clarified its ambivalent and disruptive meaning in postcolonial criticism. Subalternity was the most difficult concept, with 8 of 10 students (80%) reporting difficulty and only 3 students (30%) demonstrating contextual application. This indicates that students struggled to grasp the relationship between voice, power, and representation. Ambivalence occupied a middle position: 6 students (60%) found it difficult, while 5 students (50%) could connect it to mixed feelings toward theory, culture, or authority. Instructor emphasis also varied, with all three instructors emphasizing otherness and hybridity, while mimicry, subalternity, and ambivalence were emphasized by 2 of 3 instructors (66.7%). Overall, the table shows that students’ understanding was uneven across the five concepts, and that concepts grounded in personal experience were more easily understood than those requiring more abstract theoretical knowledge. Given the small qualitative sample, these percentages are used descriptively to clarify patterns rather than to make statistical generalizations.

## Discussion

The findings of this study reveal that the classroom is not a neutral container for knowledge transmission but a living arena where theoretical discourse is negotiated, contested, and redefined (Eagleton, 2008). Students’ difficulties with abstract terminology and their uneven interpretive engagement across cultural contexts demonstrate that theory does not simply descend intact into pedagogy; rather, it is filtered through linguistic, experiential, cultural, and affective mediations that reshape its meaning. In this study, such difficulty was especially evident in students’ engagement with the five focal postcolonial concepts: otherness, hybridity, mimicry, subalternity, and ambivalence. These concepts did not produce the same level of accessibility. As Table 4 shows, otherness and hybridity were more readily understood because students could connect them with marginality, cultural mixing, bilingual experience, and identity negotiation. By contrast, mimicry, subalternity, and ambivalence required deeper historical and theoretical explanation because they involved more complex questions of imitation, voice, representation, authority, and unstable identification. This suggests that conceptual difficulty in postcolonial pedagogy is not only a matter of abstract vocabulary, but also a matter of how far students can connect these terms to their prior knowledge, cultural experience, and understanding of colonial power. Related research on Japanese university students has similarly shown that learners perceive cognitive and metacognitive vocabulary-learning strategies as important and useful (Ueno and Takeuchi, 2023).

A particularly important finding concerns students’ uneven understanding of the basic tenets of postcolonial criticism. Before engaging with the five focal concepts, students did not enter the classroom with the same level of theoretical background. Some students understood postcolonial criticism as a critical tradition rooted in anti-colonial resistance and in challenges to Western, white, Eurocentric universalism, especially through the work of Fanon and Said. However, other students treated it mainly as a Western academic framework being applied to non-Western or non-colonial literary contexts. This uneven background knowledge helps explain why Russian students often questioned the applicability of postcolonial theory and why one Russian participant remarked that “this theory feels too Western, and I do not think it really applies to Russian literature.” Such resistance should not be interpreted simply as rejection of postcolonial criticism. Rather, it reveals a gap in students’ understanding of the theory’s intellectual genealogy and political purpose.

This finding indicates that conceptual difficulty was not only linguistic or pedagogical, but also genealogical. When students did not fully understand that postcolonial criticism developed as a critique of colonial domination, racialized knowledge hierarchies, and Eurocentric claims to universality, they could misrecognize the theory as another Western theoretical import rather than as a framework designed to challenge Western epistemic dominance. This was particularly evident among students who viewed postcolonial criticism as culturally distant from their own national literary traditions. In such cases, resistance emerged from a double misunderstanding: first, the assumption that postcolonial theory belongs only to formerly colonized societies; and second, the assumption that because the theory circulates through Anglophone academic discourse, it is itself a form of Western intellectual authority. Therefore, before students are asked to apply difficult concepts such as subalternity or ambivalence, instructors need to introduce the anti-colonial and anti-Eurocentric foundations of postcolonial criticism more explicitly.

Pedagogical translation emerges from these findings as a vital mechanism through which theory becomes actionable. The localization of abstract ideas through contextual texts, comparative examples, and dialogic teaching practices demonstrates that meaning is generated in translation rather than lost in it. Teachers’ deliberate framing of otherness, hybridity, mimicry, subalternity, and ambivalence through regionally familiar examples enabled students to anchor theoretical abstraction in lived cultural narratives. This process supports Redondo-Olmedilla’s (2023) argument that postcolonial theory acquires renewed vitality when adapted across diverse epistemic traditions. However, the findings also show that localization alone is insufficient. If localization is not accompanied by historical grounding, students may understand the concepts only as flexible interpretive tools while missing their critical connection to colonial power, resistance, and epistemic injustice. In pedagogical terms, translation should therefore not mean simplification alone; it should function as a historically informed re-theorization that makes abstract concepts accessible without detaching them from their political foundations.

The different levels of understanding shown in Table 4 further clarify how each concept entered classroom reception. Otherness was the most accessible concept, with 7 of 10 students (70%) showing contextual application, because students could link it to experiences of exclusion, marginality, and outsider positioning. Hybridity was also relatively accessible, with 6 students (60%) applying it through examples of cultural mixing, bilingual learning, and identity negotiation. Mimicry, however, was often misunderstood as simple imitation; 7 students (70%) reported difficulty before classroom explanation helped them recognize its ambivalent and potentially disruptive meaning. Subalternity was the most difficult concept, with 8 students (80%) reporting difficulty and only 3 students (30%) showing contextual application, suggesting that students struggled to grasp the relationship between voice, representation, and structural power. Ambivalence occupied an intermediate position, with 6 students (60%) reporting difficulty and 5 students (50%) applying it to mixed feelings toward theory, culture, or authority. These patterns indicate that students were more likely to grasp concepts connected to personal or observable experience, while concepts requiring historical, political, and theoretical abstraction demanded more sustained pedagogical support.

Moreover, the comparative nature of the classrooms, spanning Malaysian, Chinese, Japanese, South Korean, and Russian contexts, revealed that pedagogy itself becomes a form of comparative epistemology. When students encountered unfamiliar concepts within shared discussion, they learned not only from the text but also from one another’s interpretive positions. Malaysian and Chinese students tended to connect otherness and hybridity with identity negotiation, marginal positioning, and cross-cultural learning. Japanese and South Korean students often showed ambivalence: they recognized the analytical value of postcolonial concepts, but also questioned whether these concepts could fully explain their own literary and cultural traditions. Russian students showed stronger resistance, particularly when they understood postcolonial criticism as an external Western framework rather than as a critique of Western, white, Eurocentric universalism. These differences suggest that student reception is shaped not simply by nationality, but by the interaction between cultural background, prior theoretical knowledge, and perceived relevance of the concepts.

At the same time, students’ responses illuminated the contested universality of postcolonial theory. Some participants embraced otherness and hybridity as reflective mirrors through which to explore identity formation and global belonging, while others questioned the applicability of these concepts to contexts perceived as historically detached from colonial domination. This contested reception does not weaken the value of postcolonial criticism; instead, it reveals the pedagogical importance of teaching theory as historically situated and open to contextual reinterpretation. Postcolonial theory should not be presented as a universal grammar that can be mechanically applied to all literary texts. Rather, it should be taught as a critical framework whose meanings change when they travel across linguistic, national, and institutional contexts. In this sense, students’ resistance becomes pedagogically productive when it encourages teachers to clarify what postcolonial criticism critiques, where it comes from, and how it can be adapted without becoming detached from its anti-colonial purpose.

The data further suggest that the multicultural classroom operates as a contact zone, where theoretical meaning is co-constructed through interaction rather than predetermined by the curriculum. Students’ peer dialogs often bridged linguistic and cultural gaps, transforming misunderstanding into intellectual inquiry. Through explanation, paraphrase, disagreement, and comparison, learners collectively enacted the very dynamics of cultural translation that postcolonial theory seeks to describe. For example, peer discussion helped students move from literal understandings of mimicry as mere imitation toward more nuanced interpretations of imitation as both resemblance and resistance. Similarly, classroom dialog allowed subalternity to be approached not only as an abstract term, but as a question of who can speak, who is heard, and who is represented in literary and academic discourse. Ambivalence also became clearer when students related it to their own mixed responses toward imported theories, dominant cultural norms, or unfamiliar literary traditions. This dialogic process aligns with the ethos of decolonial pedagogy, in which learning arises through reciprocal engagement rather than hierarchical transmission.

Viewing the classroom as an interpretive community offers a powerful redefinition of pedagogy itself. When students articulate their own positions in relation to theoretical discourse, they participate in shaping the epistemic horizon of the learning space. The interplay between consensus and contestation becomes an essential pedagogical resource, sustaining intellectual vitality. However, the instructor’s role remains crucial. The findings suggest that instructors need to combine four forms of support: conceptual clarification, historical grounding, local literary examples, and dialogic application. Conceptual clarification helps students distinguish, for example, mimicry from simple imitation and ambivalence from ordinary uncertainty. Historical grounding helps students understand the anti-colonial and anti-Eurocentric roots of postcolonial criticism. Local examples make abstract theory meaningful, while dialogic application allows students to test, question, and reinterpret concepts through cross-cultural discussion. In this sense, postcolonial pedagogy is performative: it materializes the very principles of hybridity, negotiation, and multiplicity that it theorizes.

Ultimately, these findings affirm that the pedagogical life of postcolonial theory lies in its dialogic circulation rather than in its textual stability. The classroom becomes a site of ongoing epistemic renewal, where theoretical abstraction acquires relevance through interaction, disagreement, and contextual reinterpretation. Teaching postcolonial theory is therefore not merely an academic exercise but a form of cultural practice that cultivates critical literacy, empathy, and reflexivity. However, such teaching must begin from conceptual consistency and historical grounding. The five focal concepts—otherness, hybridity, mimicry, subalternity, and ambivalence—need to be introduced not as isolated vocabulary items, but as interconnected tools for examining colonial power, representation, identity formation, marginal voice, and resistance to Eurocentric knowledge structures. When students understand both the conceptual meanings and the political genealogy of these terms, postcolonial criticism can move beyond abstract discourse and become a meaningful framework for intercultural literary education. Such a reconceptualization positions the classroom as both a testing ground for theory and a generative space for decolonizing thought.

## Conclusion

This study has examined how postcolonial theory is pedagogically translated into multicultural classrooms and how students from diverse national and cultural backgrounds engage with its abstract vocabulary. By focusing on students and teachers from Malaysia, Japan, South Korea, Russia, and China, the research revealed that the comprehension of theory is not an isolated intellectual task but a socially situated and culturally mediated process. The classroom thus emerges as a dialogic arena in which theory is constantly interpreted, contested, and redefined through interaction. Rather than a stable framework imposed from above, postcolonial discourse becomes an evolving practice of negotiation, its meanings refracted through the lived experiences and interpretive repertoires of learners.

The findings underscore that pedagogy occupies a dual and often paradoxical role: it serves to make theory intelligible through simplification and contextualization, yet it also exposes the inherent instability of theoretical universality. When abstract concepts such as *hybridity*, *otherness*, or *subalternity* are localized and rearticulated, they acquire new relevance but also risk dislocation from their original theoretical matrix. These dynamics reveals that the pedagogical life of postcolonial theory depends on its flexibility, its capacity to circulate across languages, traditions, and interpretive communities without losing its critical edge. The classroom, in this sense, is not a derivative site of theory but a primary locus where theory is reconstituted as lived epistemology.

Pedagogically, the study contributes to a growing body of scholarship that situates literary theory within the ethics of intercultural learning. Localization and dialogic teaching emerge as essential strategies for rendering theoretical abstraction meaningful, while peer interaction functions as a catalyst for comparative insight. Students’ encounters with postcolonial concepts illustrate that understanding arises not from didactic explanation but from the collective labor of interpretation. Through these exchanges, learners cultivate a form of critical literacy that is both analytical and relational, an ability to read texts and theories as contested cultural artifacts situated within networks of power and history.

The study also demonstrates that resistance and ambivalence are not signs of pedagogical failure but indicators of critical engagement. Students’ hesitation to fully adopt postcolonial paradigms, especially those from contexts less shaped by colonial legacies, signal an awareness of theoretical limits and the necessity of contextual adaptation. Such resistance invites educators to reconceptualize teaching not as the affirmation of theoretical coherence but as an invitation to intellectual plurality. A decolonial pedagogy, therefore, must not aim to universalize postcolonial theory but to sustain spaces where its assumptions can be questioned, hybridized, and reimagined. In doing so, education becomes a site of epistemic justice and intercultural dialog.

Ultimately, this research affirms that the vitality of postcolonial criticism lies in its pedagogical transformation. When taught in multicultural classrooms, theory ceases to be a static body of knowledge and becomes a performative practice that mirrors the complexities of global modernity. The act of teaching itself becomes an extension of postcolonial inquiry, an exploration of how knowledge travels, mutates, and takes root in diverse epistemic soils. Future investigations might extend this inquiry through comparative, longitudinal, and multilingual frameworks to trace how sustained engagement with postcolonial ideas reshapes students’ intellectual identities. In doing so, scholars and educators can continue to reimagine the classroom as a crucible for decolonizing thought and as a generative space where theory and life converge.

## Data Availability

The raw data supporting the conclusions of this article will be made available by the authors, without undue reservation.
